# Supplementary material for: Dynamic transcriptomic profiles of zebrafish gills in response to zinc supplementation
Source: BMC Genomics. 2010 Oct 11;11:553. doi: 10.1186/1471-2164-11-553 (PMC3091702; doi:10.1186/1471-2164-11-553)
Supplement: Additional file 2 — Interactive Direct Interaction Network representing the molecular interactions between zinc, copper, iron, calcium and proteins encoded by transcripts changed by zinc supplementation. Mini web-site containing index.html and hyperlinked pages in subdirectory describing a Direct Interaction Network automatically generated based on curated interactions contained within the proprietary PathwayArchitect database. Ovals represent proteins and the circles symbolize metal ions. Objects are coloured by their abundance in zebrafish at the time-point they were significantly different from the control is a scale from -4 fold (dark green) to +4 fold (dark red). Where significant differences were found at more than one time-point, the colour overlay shows expression at the first instance. Dark blue squares denote 'binding', and light blue squares 'expression'; green squares stand for 'regulation', green diamonds for 'metabolism', and green circles for 'promoter binding'. Arrow heads indicate directionality of the interaction where annotated. All nodes and edges can be further interrogated by selecting the relative area of the image. [file 1471-2164-11-553-S2.zip › PathwayArchitect Zn xs DIN/433180.html]

# BINDING:

|  |  |
| --- | --- |
| Type | BINDING |
| Effect | None |


---

|  |  |
| --- | --- |
| Score | 0 |


---

|  |  |
| --- | --- |
| Reference Count | 9 |


---

|  |  |
| --- | --- |
| Mechanism | Unknown |


---

|  |  |
| --- | --- |
| Reference:0 || Sentence | "Prevention of Bax-VDAC interactions by a microinjection of anti-VDAC antibody effectively prevented hepatocyte apoptosis by ethanol." |
| PMID | 15044178 |
| Year | 2004 |
| Species | Rat |
| Journal | Am J Physiol Gastrointest Liver Physiol |
| RefScore | 1 |
| Source | PArchNLP |
  |
|  |  |


---

|  |  |
| --- | --- |
 Reference:1 || Sentence | "In conclusion, these data suggest that Bax translocation from the cytosol to mitochondria leads to the subsequent formation of a Bax-VDAC complex that plays a crucial role in acute ethanol-induced hepatocyte apoptosis." |
| PMID | 15044178 |
| Year | 2004 |
| Species | Rat |
| Journal | Am J Physiol Gastrointest Liver Physiol |
| RefScore | 1 |
| Source | PArchNLP |
  ||  |  |


---

|  |  |
| --- | --- |
 Reference:2 || Sentence | "A coimmunoprecipitation study revealed that Bax and Bak interacted with the voltage-dependent anion channel, which is a component of PT pores." |
| PMID | 9843949 |
| Year | 1998 |
| Species | Rat |
| Journal | Proc Natl Acad Sci U S A |
| RefScore | 1 |
| Source | PArchNLP |
  ||  |  |


---

|  |  |
| --- | --- |
 Reference:3 || Sentence | "In the present study the effect of nimesulide (NIM), a specific COX-2 inhibitor, on apoptosis and interactions between BCL-2 family death promoters BAX and BID and BAX and VDAC-1 were examined in human colon adenocarcinoma COLO 205 cells." |
| PMID | 12439336 |
| Year | 2002 |
| Species | Human |
| Journal | Anticancer Drugs |
| RefScore | 1 |
| Source | PArchNLP |
  ||  |  |


---

|  |  |
| --- | --- |
 Reference:4 || Sentence | "Bax binds to the voltage-dependent anion channel (VDAC)." |
| PMID | 11751859 |
| Year | 2001 |
| Species | Human |
| Journal | J Biol Chem |
| RefScore | 0 |
| Source | PArchNLP |
  ||  |  |


---

|  |  |
| --- | --- |
 Reference:5 || Sentence | "Further, in postischemic caudate putamen in vivo and in isolated brain mitochondria in vitro, the authors found enhanced heterodimerization between Bax and the mitochondrial membrane permeabilization-related proteins adenine nucleotide translocator (ANT) and voltage-dependent anion channel." |
| PMID | 11323518 |
| Year | 2001 |
| Species | Rat |
| Journal | J Cereb Blood Flow Metab |
| RefScore | 2 |
| Source | PArchNLP |
  ||  |  |


---

|  |  |
| --- | --- |
 Reference:6 || PMID | 9843949 |
| Species | Rat |
| MINT interaction detection method | pull down |
| Source | MINT |
  ||  |  |


---

|  |  |
| --- | --- |
 Reference:7 || PMID | 9843949 |
| Species | Rat |
| MINT interaction detection method | coimmunoprecipitation |
| Source | MINT |
  ||  |  |


---

|  |  |
| --- | --- |
 Reference:8 || PMID | 9843949 |
| Species | Rat |
| MINT interaction detection method | coimmunoprecipitation |
| Source | MINT |
  |


---

|  |  |
| --- | --- |
